# Evaluation of Factors Predicting Successful Outcome in Patients Undergoing Expectant Management of Uncomplicated Acute Appendicitis

**DOI:** 10.7759/cureus.48687

**Published:** 2023-11-12

**Authors:** Bhrigu Prajapat, Rajkumar Chejara, Mahesh K Mittal, Arya S V, Dheer S Kalwaniya

**Affiliations:** 1 General Surgery, Vardhman Mahavir Medical College and Safdarjung Hospital, New Delhi, IND; 2 Radiology, Subharti Medical College, Meerut, IND

**Keywords:** appendix diameter, pulse rate, pr, ad, crp, tlc, uncomplicated acute appendicitis, nom, expectant management

## Abstract

Background: Expectant management, or non-operative management (NOM), with standalone intravenous antibiotic therapy, has emerged as an effective alternative to appendectomy for the treatment of uncomplicated acute appendicitis. Various clinical, biochemical, and radiological factors have been implicated in predicting the success or failure of outcomes. Therefore, it is important to identify patients at the onset who are likely to have successful outcomes for conservative management of uncomplicated acute appendicitis.

Methods: We prospectively enrolled 85 surgical patients diagnosed with uncomplicated acute appendicitis in our study. On admission, clinical features such as duration of symptoms, pulse rate (PR), history of fever within 24 hours of admission, modified Alvarado score and adult appendicitis score, biochemical parameters such as C-reactive protein (CRP), and hematological parameters such as total leukocyte count (TLC) were recorded. Radiological imaging of patients, namely ultrasonography and contrast-enhanced CT abdomen to evaluate appendix diameter and mural enhancement, was also undertaken. The outcome of expectant management for these patients (success or failure) was recorded, and the above-mentioned factors were evaluated to find a possible correlation with successful expectant management.

Results: We found that among 85 patients, 77.6% had a successful NOM of appendicitis, whereas 22.4% had a failed NOM of appendicitis. The duration of symptoms, pulse rate, fever within 24 hours of admission, TLC, percentage of neutrophils, CRP level, appendix diameter, modified Alvarado score, and adult appendicitis score^ ^were found to be statistically significant predictors of successful NOM of appendicitis according to univariate analysis. According to multivariable analysis, pulse rate and appendix diameter value were found to be statistically significant predictors of successful NOM of appendicitis. With each beat per minute (bpm) increase in pulse rate, there was a 0.30% decrease in the probability of a successful NOM of appendicitis. With each mm increase in appendix diameter, there was an 82% decrease in the probability of a successful NOM of appendicitis.

Conclusion: From our study, it can be concluded that patients who met the following criteria, i.e., duration of symptoms before presenting to surgical emergency less than two days, presence of fever within 24 hours of presenting to surgical emergency, pulse rate >90 bpm, TLC >12000 cells/dL, CRP >20 mg/L, appendix diameter >10 mm, modified Alvarado score ≥ 9, and adult appendicitis score ≥ 18, have a higher probability of failure of NOM and hence should be excluded from expectant management.

## Introduction

Acute appendicitis is one of the most common causes of lower abdominal pain, leading patients to come to the emergency department. It is also one of the most common diagnoses made in young patients who are admitted to the hospital with an acute abdomen, as shown by Cervellin et al. [1]. Appendectomy, whether open or laparoscopic, is the gold standard and least controversial treatment of acute, uncomplicated appendicitis to date. Certain population-based studies have shown some serious long-term risks following surgery for acute appendicitis, such as small bowel obstruction, requiring operation in 1.3% of patients within 30 years, and a 30-day mortality rate of 0.24% with an increased standard mortality ratio [2-4].

Expectant management with intravenous antibiotic therapy alone has emerged as an effective alternative to appendectomy for the treatment of uncomplicated acute appendicitis [5,6]. It is important to identify patients at the onset of the disease who are likely to have successful outcomes for conservative management of uncomplicated acute appendicitis. There is a paucity of studies that look into factors that predict a successful outcome in a patient with uncomplicated acute appendicitis undergoing non-operative management (NOM).

## Materials and methods

This is a hospital-based prospective observational study conducted from October 2020 to March 2022, approved by the Institutional Ethics Committee of Vardhman Mahavir Medical College and Safdarjung Hospital, New Delhi, India (approval no. IEC/VMMC/SJH/Thesis/2020-11/CC-130). A total of 85 patients admitted with uncomplicated acute appendicitis in the Department of Surgery at Vardhman Mahavir Medical College and Safdarjung Hospital were studied.

Inclusion and exclusion criteria

All patients were at least 18 years of age and diagnosed with uncomplicated acute appendicitis based on clinical symptoms and signs and confirmed radiologically on ultrasound and/or contrast-enhanced CT of the abdomen. All patients less than 18 years of age, pregnant women, and immunocompromised patients were excluded from the study.

Data collection

The 85 patients who presented with complaints of right lower quadrant pain with symptoms and signs strongly suggestive of uncomplicated acute appendicitis that was confirmed on ultrasonography, were included. The modified Alvarado score and adult appendicitis score were calculated, and a contrast-enhanced CT of the abdomen was done to confirm appendicular diameter.

Laboratory measurements

Standardized data collection forms were used to collect information pertaining to the demographic, clinical, and laboratory parameters of the study subjects. An automated hematology analyzer measured the CBC as well as the total leukocyte count (TLC) and percentage of neutrophils. An automated protein analyzer, the MISPA i3 (Agappe Diagnostics Ltd., Ernakulam, KL, India), was used for the quantitative measurements of C-reactive protein (CRP). Blood samples were analyzed on day 1, i.e., at the initial presentation to the hospital.

Definition of a failed NOM

All the patients were given an intravenous antibiotic course with IV ceftriaxone 1 gm/12 hourly and IV metronidazole 500 mg/8 hourly, the antibiotic therapy used by previous researchers [7]. Treatment failure was defined as either a lack of improvement in the patient or clinical progression of the acute appendicitis indicated by an increase in TLC, pulse rate, presence of persistent fever, worsening of per abdominal signs, and increasing CRP that necessitated an emergency appendectomy while attempting expectant management in the admitted patient within 48 hours of his or her admission.

## Results

Among 85 patients, 77.6% had successful expectant (non-operative) management of appendicitis, 22.4% had failed expectant management, 77.5% had successful NOM of appendicitis, and among 45 males, 77.8% had successful NOM of appendicitis. However, the difference in the proportion of successful NOM of appendicitis according to gender was statistically not significant (p > 0.05). The evaluated factors that were observed to be statistically significant (p < 0.05) in predicting successful NOM, namely pulse rate, TLC, CRP levels, mean appendix diameter on ultrasonography and CT, modified Alvarado score value, and adult appendicitis score value, are detailed in Table 1.

**Table 1:** Comparison between successful NOM and failed NOM based on different parameters which were statistically significant NOM: Non-operative management

Variables	Successful NOM	Failed NOM	p-value
Median (IQR) pulse rate (bpm)	88 (80,91)	98 (91,101)	0.001
Median (IQR) total leukocyte count (cells/dL)	9600(8250,11075)	12100(10000,13650)	0.001
Median (IQR) C-reactive protein level (mg/L)	14 (10,20)	28 (18.5,38)	<0.001
Mean (±SD) appendix diameter on ultrasonography (in mm)	8.92 ± 1.00	10.31 ± 0.90	<0.001
Mean (±SD) appendix diameter on CT scan (in mm)	9.01 ± 0.97	10.52 ± 1.01	<0.001
Median (IQR) modified Alvarado score	7 (7,8)	9 (8,9)	<0.001
Median (IQR) adult appendicitis score	16 (15,18)	19 (17,22)	<0.001

The factors that were observed to be statistically not significant (p-value > 0.05) in predicting successful NOM, namely age, gender, and mural enhancement on CT, are summarized in Table 2.

**Table 2:** Comparison between successful NOM and failed NOM based on parameters that were observed to be statistically not significant NOM: Non-operative management

Variables	Successful NOM	Failed NOM	p-value
Median (IQR) age (in years)	26 (21, 33)	26 (23, 33)	0.661
Gender	Female: 31 (77.5%)	9 (22.5%)	1.000
	Male: 35 (77.8%)	10 (22.2%)	
Mural enhancement	Yes: 20 (76.9%)	6 (23.1%)	1.000
	No: 46 (78.0%)	13 (22.0%)	

According to multivariable analysis, as shown in Table 3, pulse rate and appendix diameter were found to be statistically significant predictors of successful NOM of appendicitis. With each bpm increase in pulse rate, there was a 0.30% decrease in the probability of a successful NOM of appendicitis (adjusted odds ratio (aOR) 0.70, 95% CI 0.47-0.87). With each mm increase in AD, there was an 82% decrease in the probability of a successful NOM of appendicitis (aOR 0.18, 95% CI 0.03-0.56).

**Table 3:** Univariate and multivariable logistic regression to assess the predictors of successful NOM of appendicitis NOM: Non-operative management, cOR: Crude odds ratio, aOR: Adjusted odds ratio

Variables	Univariate model		Multivariable model	
	cOR (95% CI)	p-value	aOR (95% CI)	p-value
Duration of symptoms (in days)	5.39(1.69-24.4)	0.010	9.49 (0.67-394.75)	0.137
Pulse rate (bpm)	0.81(0.72-0.89)	<0.001	0.70 (0.47-0.87)	0.013
Fever within 24 hours of admission	0.23 (0.05-0.75)	0.027	0.35 (0.01-5.07)	0.456
Total leukocyte count (cells/dL)	0.9996 (0.9993-0.9998)	<0.001	0.9999 (0.9993-1.0005)	0.797
C-reactive protein level (mg/L)	0.86 (0.79-0.92)	<0.001	0.79 (0.56-0.95)	0.065
Appendix diameter (in mm)	0.22 (0.09-0.42)	<0.001	0.18 (0.03-0.56)	0.011
Modified Alvarado score	0.33 (0.16-0.61)	<0.001	2.44 (0.33-24.97)	0.385
Adult appendicitis score	0.65 (0.51-0.79)	<0.001	0.63 (0.25-1.28)	0.247

**Figure 1:**
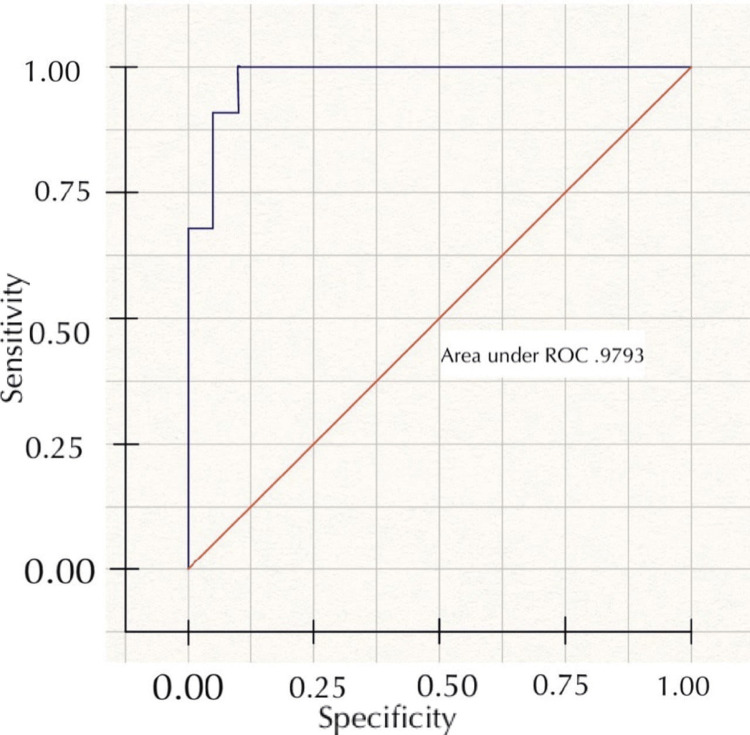
ROC curve to assess predictive accuracy for multivariable model in predicting successful NOM of appendicitis ROC: Receiver operating characteristic, NOM: Non-operative management

Figure 1 shows the area under the receiver operating characteristic curve for the multivariable model in predicting the successful NOM of appendicitis, which was found to be 0.9793 (0.945-1.000).

## Discussion

There have been some studies in Western countries regarding the identification and evaluation of factors that successfully predict which patients will experience successful expectant or NOM for uncomplicated acute appendicitis with intravenous antibiotics not requiring an emergency appendectomy [8-13]. We conducted a study for the comprehensive evaluation of certain clinical, biochemical, and radiological factors that can effectively predict success in patients undergoing expectant or NOM for uncomplicated acute appendicitis.

In our study, it was observed that patients who had a successful expectant or NOM had a longer median duration of symptoms before presenting to a surgical emergency of two or more days, whereas patients who had a shorter median duration of symptoms were more likely to have a failure of expectant management, which was found to be statistically significant (p <0.05). The study by Tyler et al. concludes that a longer duration of symptoms before admission is related to successful expectant management of acute appendicitis, as they observed that the mean duration of symptoms for successful expectant management was 44 hours, which was longer than the average duration of 24 hours for failed NOM, which was found to be statistically significant with a p-value of 0.023 [8]. Similar results were observed by Walker et al., who concluded that multivariate analysis revealed that a longer duration of symptoms reduced the likelihood of failure [9]. This is also in line with the traditional knowledge that a longer duration of symptoms equates to more severe disease and, hence, is more likely to fail NOM.

In this study, the patients with successful expectant or NOM had a lower median pulse rate (88) and beats per minute (80,92), whereas, in patients with failure of expectant or NOM, the median pulse rate was observed to be considerably higher (98 beats per minute (91,101)) which was statistically significant (p < 0.05). However, in the previous research by Tyler et al., the pulse rate was observed to be insignificant as per their analysis; this difference may be due to the fact that in our study only cases of uncomplicated acute appendicitis were included, whereas there was no such inclusion factor in the aforementioned study [8]. However, the pre-disease or resting pulse rate of all the patients was not known, which is one of the limitations of our study.

It was observed that among 52 patients who had a fever within 24 hours of presenting to the surgical emergency, 69.2% had successful expectant management, whereas the 33 patients presenting without fever had a success rate of 90.9%. This was statistically significant and is comparable to the observations made by previous researchers, as in the study by Imaoko et al., where the presence of fever (>37.40 C) is significantly associated with an increase in failure of NOM [10].

It was observed that the patients with successful NOM had comparatively lower TLC (IQR median 9600 cells/dL) and a lower mean percentage of neutrophils on differential leukocyte count (DLC) (76.7%) compared to the other group of patients with failed NOM, who had higher TLC (IQR median 12100 cells/dL) and a higher percentage of neutrophils on DLC (mean 82%), which was statistically significant. It is similar to the conclusions made by certain previous researchers: the study conducted by Jeanette et al. observed that patients with a TLC of less than 12000 cells/dL had an 89% incidence of success in expectant management with a p-value of <0.05 [11].

The CRP levels were observed to be a significant individual factor in univariate analysis for predicting successful NOM, as the levels were significantly lower in patients with successful expectant management (IQR median 14 mg/L), whereas their value was higher in patients with failed expectant management (IQR median 28 mg/L). This observation and inference are in agreement with previous researchers, Shindoh et al., who inferred that a CRP value > 40 mg/L is a statistically significant predictor of failure of expectant management [12]. Previous research by Okus et al. infers that the optimum CRP level cut-off for unsuccessful expectant management was 80.8 mg/L [13]. This high value is also attributable to the fact that they included complicated appendicitis in their study as well, and most of those patients had a higher mean CRP level, which could’ve skewed the values obtained by them. Patients with the aforementioned findings, namely the presence of fever, a higher TLC count, and higher CRP levels, had poor success rates, as these represent a more severe and possibly complicated disease that is less amenable to resolution with IV antibiotics alone and may be better treated with surgical management by laparoscopic or open appendectomy.

The appendix diameter was observed to be a significant predictor of success in expectant management, as the patients with a comparatively smaller appendix diameter (mean 9 mm) were observed to have successful expectant management. This inference is similar to that of the previous researchers, as a comparable result is seen in a study by Koike et al., where they concluded that of the 19.2% of patients with failure of expectant management of uncomplicated acute appendicitis, a mean appendiceal diameter of >9 mm was observed to be statistically significant with a p-value <0.05 [14]. The meta-analysis by Kim et al., which evaluates 23 studies and 10 CT parameters, concludes that only periappendiceal fat stranding showed high sensitivity (94%) but low specificity (40%) in diagnosing patients with complicated appendicitis that required surgery [15]. The study by Tyler et al. also observes that a median diameter of 9 mm is associated with successful expectant management [8].

The above result is attributable to the fact that in appendicitis, with progressive distension of the appendix, impairment of the venous drainage occurs, which in turn leads to mucosal ischemia and mucosal ulceration, and this ultimately leads to full-thickness ischemia, which is not amenable to complete resolution by NOM alone. Furthermore, the onset of full-thickness ischemia may lead to necrosis, which in turn may lead to appendiceal perforation, which is better treated with appendectomy. According to the multivariable analysis of our observations, only two factors, i.e., lower pulse rate and lower appendix diameter, were found to be statistically significant predictors of successful expectant management of uncomplicated acute appendicitis.

Limitations of the study

There are certain limitations to this study and the given outcomes, as the sample size was only 85 patients and the follow-up period of this study was only two days. As mentioned previously, the pre-disease or resting pulse rate of the patients was not known. Thus, the absolute increase in pulse rate from resting pulse rate is not known.

## Conclusions

From this study, it can be concluded that patients who meet the following criteria are more likely to fail expectant management and thus should not be considered for the NOM of uncomplicated acute appendicitis. These include the duration of symptoms before presenting to a surgical emergency <2 days, the presence of fever within 24 hours of presenting to a surgical emergency, a pulse rate >90 beats per minute, TLC >12000 cells/dL, CRP >20 mg/L, appendix diameter >10 mm, modified Alvarado score ≥ 9, and adult appendicitis score ≥ 18.
